# Enteral Nutrition Support to Treat Malnutrition in Inflammatory Bowel Disease

**DOI:** 10.3390/nu7042125

**Published:** 2015-03-25

**Authors:** Roberta Altomare, Giuseppe Damiano, Alida Abruzzo, Vincenzo Davide Palumbo, Giovanni Tomasello, Salvatore Buscemi, Attilio Ignazio Lo Monte

**Affiliations:** 1 School in Surgical Biotechnology and Regenerative Medicine, School of Medicine, School of Biotechnology, University of Palermo, Via del Vespro 129, Palermo 90127, Italy; E-Mails: roberta.altomare@unipa.it (R.A.); alida.abruzzo@unipa.it (A.A.); vincenzodavide.palumbo@unipa.it (V.D.P.); salvatore.buscemi@unipa.it (S.B.); 2 AUOP “P.Giaccone”, Universitary Hospital, Via del Vespro 129, Palermo 90127, Italy; E-Mails: guise.damiano@gmail.com (G.D.); giovanni.tomasello@unipa.it (G.T.); 3 GENURTO Department, School of Medicine and Biotechnology, University of Palermo, Via del Vespro 129, Palermo 90127, Italy

**Keywords:** enteral feeding, malnutrition, nutrients supplementation, inflammatory bowel disease, tube feeding, home enteral nutrition

## Abstract

Malnutrition is a common consequence of inflammatory bowel disease (IBD). Diet has an important role in the management of IBD, as it prevents and corrects malnutrition. It is well known that diet may be implicated in the aetiology of IBD and that it plays a central role in the pathogenesis of gastrointestinal-tract disease. Often oral nutrition alone is not sufficient in the management of IBD patients, especially in children or the elderly, and must be combined with oral supplementation or replaced with tube enteral nutrition. In this review, we describe several different approaches to enteral nutrition—total parenteral, oral supplementation and enteral tube feeding—in terms of results, patients compliance, risks and and benefits. We also focus on the home entaral nutrition strategy as the future goal for treating IBD while focusing on patient wellness.

## 1. Introduction

Enteral nutritional therapy aims to maintain or restore the nutritional status of individuals who fail to maintain a sufficient oral intake, despite having a fully or partially functioning gastrointestinal tract. Its administration is related to the reduction of infectious complications and maintenance of the integrity of intestinal flora [1]. Lesions of the jaw and central nervous system, anorexia, cancer, hypermetabolic conditions such as burns and severe infections, and, above all, IBD are examples for enteral nutrition indicators [1,2].

The term IBD includes at least three clinical conditions: ulcerative colitis (UC), Crohn’s disease (CD) and indeterminate colitis (IC). IBD can occur at all ages and in both genders. Patients usually complain of severe diarrhea, abdominal pain, weight loss and fever, which severely interfere with their quality of life. Understanding the aetiology of IBD is still controversial as it is a multifactorial pathology; it likely depends on an interaction between susceptible genes and environmental factors leading to an abnormal chronic immunological response that causes tissue injury with bowel inflammation and ulceration [2,3].

It is known that the diet plays a role in the pathogenesis of IBD, as has been demonstrated by exclusion diets in several studies [4]. However, it is possible that some nutrients act either as antigens or have therapeutic effects in the intestinal mucosa [2].

In both UC and CD, nutritional deficiencies of various severities are often found. These include protein-energy malnutrition and various vitamin, mineral and trace element deficiencies [5]. Malnutrition in IBD depends on several factors such as inflammatory processes, impaired intestinal absorption, nutrients loss through the inflamed and ulcerated gut, bacterial overgrowth, medical management with steroids or imposed dietary restrictions [2,5].

These observations suggest that nutritional support may be the best approach to treat IBD instead of using drugs, as it could control inflammation and treat malnutrition without side effects.

The first attempt to use enteral nutrition as primary therapy in IBD was made with amino acid-based elemental diets in patients with active CD. The concept was that providing amino acids (elemental diets) instead of intact proteins (polymeric diets) would decrease the intestinal antigenic load in the gut lumen, which in turn would decrease the chances of triggering or maintaining the abnormal or up-regulated inflammatory bowel response. Elemental diets have been shown to decrease intestinal permeability and diminish the excretion of pro-inflammatory cytokines in the stools in CD patients [6,7].

One of the most important goals of nutritional therapy should be to prevent and treat undernutrition, improving the growth and development of children and adolescents using total parenteral nutrition (TPN), enteral nutrition (EN), or by correcting micronutrient deficiency [8,9].

## 2. Feeding Methods and Patients Management

Sip feeding, tube feeding and parenteral nutrition have all been tested and found to be comparably effective. The selection of the form of nutritional therapy is therefore dependent on secondary factors such as cost, side effects and inconvenience for the patient. Based on these considerations, enteral tube feeding is the primary choice for nutritional therapy of an active phase of Crohn’s disease. The mechanism by which nutritional therapy affects the active phase of Crohn’s disease is not clear. Two hypotheses have been discussed: (1) bowel rest with reduction of luminal bacteria and antigens leading to a decrease in inflammation; and (2) induction of anabolism changing the immune reaction and thereby reducing inflammation. It is likely that both mechanisms contribute.

Tube feeding is the preferred nutritional therapy for the active phase of Crohn’s disease. Sip feeding is similarly effective, provided that patients drink enough from the diet to meet their requirements. This is difficult to achieve, however. Sip feeding is hampered by a high rate of non-compliance due to the poor palatability of the diets. Therefore, until more palatable diets are available, patients should be offered tube feeding as the treatment of first choice [10].

### 2.1. Total Parenteral Nutrition (TPN)

TPN, although it is the only way of putting the bowel at complete rest, has not been shown to be superior to tube feeding. It may be indicated in some restricted cases, such as in an obstructed bowel that is not amenable to feeding tube placement beyond the obstruction, a short bowel resulting in severe malabsorption or fluid and electrolyte loss that cannot be managed enterally, severe dysmotility in which enteral feeding is impossible, a leaking intestine from high-output intestinal fistula or surgical anastomotic breakdown, in patients intolerant to EN whose feeding cannot be maintained orally, when there is an inability to access the gut for enteral feeding, and in patients undergoing IBD-related bowel surgery in the perioperative period [8,11].

Available data so far show that while artificial nutrition seems to play a primary role in the management of patients with active CD, it does not have a primary therapeutic effect in active UC and does not induce clinical remission of this type of IBD [12].

The use of TPN in the management of active CD is based on theoretical advantages including bowel rest, which could reduce the motor and transport functions of the diseased bowel; reduction of antigenic stimulation, which could eliminate the immunological response to food favored by the presence of impaired intestinal permeability; and stimulation of protein synthesis, which could lead to cell renewal and mucosal healing in the intestine [13]. Nevertheless, few controlled clinical trials have been conducted on the use of TPN to induce remission in active CD. The remission rate three months after starting TPN varied from 20% to 79%, depending on the patient population, length of TPN administration, definitions of remission or recurrence, and concomitant use of medication [14]. TPN has also been shown to achieve fistula healing in 43–63% of patients, accompanied by disease activity reduction and weight gain [15]. TPN was also associated with an increased risk of adverse events such as sepsis and cholestatic liver disease [12].

### 2.2. Enteral Nutrition (EN)

In addition to the fact that it delivers normal food, EN (with oral nutritional supplements or tube feeding) may be more useful than TPN in the management of undernourished patients with IBD. If the gut can be used safely, EN is actually the preferred feeding method for CD or UC patients who need nutritional support. The advantages of EN include its stimulatory effects on gastrointestinal structure and function as well as its reduced cost compared to parenteral feeding. Oral nutritional supplements (with 500–600 kcal·day^−1^) and/or tube feeding improve the nutritional status in adults and especially in children with CD [16]. In fact, 50% of growth-retarded CD patients cannot regain their body weight through medical therapy alone and must use enteral tube feeding [17].

Several studies have demonstrated the efficacy of EN in active CD. While EN’s mechanisms of action remain unknown, several hypotheses have been proposed, including the ability of nutrients to modulate the commensal microflora and the intestinal immune response by reducing antigen exposure. In fact, EN seems to exert a direct antiinflammatory effect on the intestinal mucosa by reducing IL-6 production and increasing insulin-like growth factor (IGF)-1 production [18]. However, EN has been proven to be effective in the treatment of the acute phase of CD, achieving remission rates from 20% to 84.2% regardless of disease location. The variability in these results may stem from differences among study populations, administration protocols and outcome assessments [12].

To date, no definitive data has been published on supplementation with oral nutritional supplements in UC patients, who should undergo enteral tube feeding only in exceptional cases [12].

#### 2.2.1. Oral Nutritional Supplementation

Patients with IBD often report concern that their diet may exacerbate their symptoms, and many modify their diet in the hope of controlling symptoms or preventing relapse [19].

This becomes a concern when patients drastically reduce or completely avoid nutritionally important foods, which may put them at risk of developing nutritional deficiencies. Most patients believe that diet influenced their disease and modify their diet. The most common behaviour is the avoidance of milk and dairy products; this dietary change resulted in reduced calcium intakes but had no apparent effect on the rate of relapse [20,21].

Moreover, the exclusion of fruit and vegetables is common (legumes 29.5%; vegetables 18% and fruit 11%) [21]. Unnecessary dietary exclusions, particularly of milk and dairy products, are a concern if initiated without appropriate nutritional or medical supervision.

Although some data suggest that dietary factors play a role in the onset and the course of IBD, recommendations other than following a healthy and varied diet cannot currently be made for most patients [22]. Prescribing a low-residue diet, low in insoluble fiber, may be advisable during acute flares of IBD, particularly in patients with stricturing CD or severe UC attacks [23]. Recently, several hypotheses about the possibility that some specific nutrients can modulate inflammation have been suggested. Specifically, the anti-inflammatory effects of *n*-3 (omega-3 fatty acids, fish oil) have been suggested to be beneficial in chronic inflammatory disorders such as inflammatory bowel disease [24]. At present, data are available mainly on the beneficial effect of *n*-3 polyunsaturated fatty acids (PUFAs) and fermentable fiber [22].

Fermentable fiber generates much less residue than insoluble fiber, and it is fermented by colonic microflora, yielding several products, such as butyrate, than can be beneficial for IBD. Butyrate is the main metabolic substrate for colonic epithelial cells, and there is *in vitro* evidence suggesting that butyrate is able to down-regulate the production of pro-inflammatory cytokines, to promote the restoration of intracellular reactive oxygen species (ROS) balance and the activation of NF-κB [25].

#### 2.2.2. Enteral Tube Feeding

The mechanism underlying a therapeutic response to enteral diets remain unclear [19]. Initially, it was thought that a low antigenic load (absence of whole protein) in the elemental diet was responsible for inducing remission—but it is now known that whole protein enteral feeds are as effective as elemental diets [26]. One of the theories that offers the most exciting possibilities concerns the potential anti-inflammatory effect of an enteral diet on the gastrointestinal mucosa [27]. This may be related to the provision of fatty acids in the feed [28] and/or the potential of the feed to alter gut flora. There is now stronger evidence that the clinical response to an enteral diet is accompanied by histological healing of the mucosa and down regulation of mucosal pro-inflammatory cytokines [27].

Enteral feeding is largely free from side effects. Minor side effects may occur and nausea and headaches may be reported, but these usually resolve after the first few days of feeding. Gradual introduction of the feed during the initial 3–4 days should limit diarrhea. Weight loss, abdominal cramps and vomiting can also occur but they usually resolve as the patient adapts to the diet. The main problems with this regime are often related to poor compliance and unpalatability. These problems may be improved by administering this therapy as part of a multidisciplinary team approach involving the medical, dietetic and nursing staff, as well as the patient and family members, and providing support and education. It is important to acknowledge that exclusive enteral nutrition can, understandably, be a demanding and difficult therapy for many patients [19].

Patients should receive 25–35 kcal·kg^−1^·day^−1^ by continuous infusion via a nasogastric tube. Infusion rates over 120 mL·h^−1^ frequently lead to diarrhea or reflux. To limit the infusion rate, nutrition should be infused 24 h a day.

It is useful to start enteral nutrition at a low infusion rate of 20 mL h^−1^ and increase this over 2–3 days to the full dose. Additionally, in many patients who are dehydrated due to severe diarrhea, electrolyte solutions have to be infused parenterally during the first few days.

Enteral nutrition should be continued for a minimum of two and preferably four weeks. The dose and duration is dependent on clinical parameters like nutritional status and decrease in disease activity. When symptoms improve, the patient may be allowed some additional food and enteral nutrition may be reduced, dovetailing with the increase in oral nutrition [10].

### 2.3. Home Enteral Nutrition (HEN)

The most common home infusion therapy today is home enteral nutrition (HEN) or home enteral tube feeding (HETF). HEN should be used in patients who cannot meet their nutrient requirements by oral intake but have a functional gastrointestinal tract, and who are able to receive therapy outside of an acute care setting. It is estimated that more than 300,000 people of all ages in the USA are receiving enteral nutrition at home, whereas in Europe, HETF in the community has also considerably increased in the last few years [29]. Epidemiologic data from UK show that, at any one time, over 19,500 patients receive HETF in the UK community, more than twice that in hospitals [30].

Several factors have contributed to the rapid growth of HEN, including increased awareness of therapeutic nutrition, developments in artificial nutrition, a higher proportion of elderly people in the population, and reduction in the number of hospital beds.

Gastrostomy and/or jejunostomy feeding tubes are frequently inserted and used for long-term home enteral nutrition support. Although insertion of these tubes is usually related to minor morbidity, their long-term use may contribute to various complications and problems which may affect quality of life and have significant economic consequences on health care use [29].

Percutaneous endoscopic gastrostomy (PEG) is actually indicated for patients requiring long-term nutritional support (>30 day) who have a functional gastrointestinal tract but insufficient oral intake of nutrients. There are three techniques for PEG tube placement: the peroral pull technique, the peroral push technique and the direct percutaneous procedure [31]. The most widely-used technique for PEG placement is the ‘‘pull’’ method introduced by Gauderer *et al.* in 1980 [32], which has replaced surgical gastrostomy as the medium- and long-term solution to enteral nutrition delivery, being safer and more cost-effective, with lower procedure-related mortality (0.5%–2%). Furthermore, tube displacement occurs less frequently than with nasogastric tubes [31].

Long-term jejunal feeding can be achieved endoscopically with jejunal tubes through the PEG (JET-PEG) and direct percutaneous endoscopic jejunostomy (DPEJ). Jejunal feeding is appropriate for patients with recurrent vomiting and/or tube feeding-related aspiration, severe gastroesophageal reflux, gastroparesis, gastric outlet obstruction, or total or partial gastrectomy [31].

A complication related to PEG tubes is the formation of gastrocolic, colocutaneous or gastrocolocutaneous fistulae, especially in IBD patients with an active disease. In contrast to the gastrocolic fistula, a fistulous passage connecting the stomach with the colon, the gastrocolocutaneous fistula is defined as an epithelial connection between the mucosa of the stomach, the colon, and the skin. Its probable etiology is the penetration of a bowel loop (mostly transverse colon) interposed between the stomach and the abdominal wall, either by inadvertent puncture during tube placement or, more commonly, due to gradual erosion of the tube into the adjacent bowel [33].

## 3. Complications of Enteral Tube Feeding

Despite the overall safety of feeding tubes, a number of complications can occur following their placement, though they are usually considered minor, including tube dislodgment, peristomal leakage, and wound infection [34,35]; also, most studies have suggested that complications are more likely to occur in elderly patients with comorbid illnesses, particularly those with an infectious process or who have a history of aspiration [36].

Nevertheless, enteral tube feeding is considered “routine” by many health care professionals involved with it, because these tubes are the most common and easiest to manage in the community. However, as tube feeding can still be a daunting thought for patients and caregivers, careful consideration should be given to predischarge planning and training. Planning for discharge on HEN should begin at the earliest opportunity and involve all the relevant health care professionals and community staff [37], while discussing with the patients and caregivers what to expect on a daily basis when administering HEN [38]. Also, training patients and/or caregivers on caring for the tube, hygiene issues, safety, and basic problem solving is of paramount importance, and they must be clear regarding arrangements for supply of feed and equipment. Consequently, by the time of discharge, patients and caregivers should be adequately trained on the various aspects of the tube feeding system, to ensure safe and effective feeding at home [29].

As tube dysfunction is the most frequent complication related to its presence, some simple measures must be taken to prevent or, at least, to decrease its incidence. For example, tubes may become clogged or occluded if not flushed with water after each feeding, or feedings may leak around the exit site of the tube if tube is too loose, if the balloon is broken, if the tract is enlarged, or the stomach too full. Consequently, the placement of the tube must be frequently checked, and it must be resecured to keep it in place [29].

Recently, it was demonstrated that using a nasal bridle can decrease inadvertent removal of nasally inserted enteral tubes and improve subsequent patient outcomes. The use of the bridles may be benefical in patients with conditions that limit the effectiveness of traditional tubes [39].

## 4. Conclusions

Enteral nutrition should be used as primary therapy in CD for a number of reasons. First, it fulfils the therapeutic criteria for use in a wide group of patients with CD, since it achieves equal or higher remission rates than some of the drugs currently used. At the same time, it is free of the aesthetic, haematologic, systemic and metabolic side effects commonly associated with steroids and other immune modulators. In addition, in some cases, mucosal healing has been demonstrated with this therapy. Second, enteral nutrition treats or prevents nutritional deficits associated with IBD. It also enhances growth and sexual development in children and adolescents and partly prevents or reverses osteopenia. Enteral nutrition should be the treatment of choice for children and adolescents, not only for the first attack but also for any relapse and as maintenance therapy. Similarly, it should be seen as the first possible treatment for elderly patients with CD in order to maintain their bone mass, since there is a strong possibility that they may have received various long-term treatments with glucocorticoids during their life. Third, enteral formulas should be tried for new onset attacks of CD at all ages to avoid steroid side effects. It should also be the first therapeutic approach in all mild to moderate acute attacks, in particular when there is small intestine or ileocolonic involvement. Finally, a further advantage of this approach is that it is a safer way of starting the treatment in patients with a possible undiagnosed abdominal abscess [2].
